# Supplementary material for: Reassessment of Viroid RNA Cytosine Methylation Status at the Single Nucleotide Level
Source: Viruses. 2019 Apr 18;11(4):357. doi: 10.3390/v11040357 (PMC6521008; doi:10.3390/v11040357)
Supplement: Supplementary file 1 [file viruses-11-00357-s001.pdf]

SUPPLEMENTARY MATERIAL

Reassessment of viroid RNA cytosine methylation status at the single nucleotide level

Francesco Di Serio[1], Enza Maria Torchetti[1], José-Antonio Daròs[2,*], Beatriz Navarro[1,*]

Figure S1 Bisulfite sequencing of - PSTVd RNAs. Bisulfite cytosine conversion generated by the standard (St) and the improved (Im) protocols of a - PSTVd fragment amplified with the primer pair PSTVd_met_5F_minus/PSTVd_minus_6R_plus. The sequences targeted by the specific primers (salmon background) and the positions of cytosines in the reference variant PSTVd-Nb (green background) are indicated on the top. Converted (T, grey background) and unconverted (C, white background) cytosines are reported for each sequenced clone, the name of which is reported on the left, together with the percentage of unconverted cytosines.

Figure S2 Bisulfite sequencing of + and - ASBVd RNAs. Bisulfite cytosine conversion generated by the standard (St) and the improved (Im) protocols of + and - PSTVd fragments amplified with the primer pairs ASBVd_met_1F_plus/ ASBVd _met_2R_plus and ASBVd _met_3F_minus/ ASBVd _met_4R_minus (panel A and panel B, respectively). The sequences targeted by the specific primers (salmon background) and the positions of cytosines in the reference variant ASBVd (green background) are indicated on the top. Converted (T, grey background) and unconverted (C, white background) cytosines are reported for each sequenced clone, the name of which is reported on the left, together with the percentage of unconverted cytosines.

Figure S3 Bisulfite sequencing of + HSVd *in vitro* transcript. Bisulfite cytosine conversion generated by the standard (St) and the improved (Im) protocols of a + HSVd fragment amplified with the primer pair HSVd_met_1F_plus/HSVd_plus_2R_plus. The sequences targeted by the specific primers (salmon background) and the positions of cytosines in the reference variant HSVd (green background) are indicated on the top. Converted (T, grey background) and unconverted (C, white background) cytosines are reported for each sequenced clone, the name of which is reported on the left, together with the percentage of unconverted cytosines.

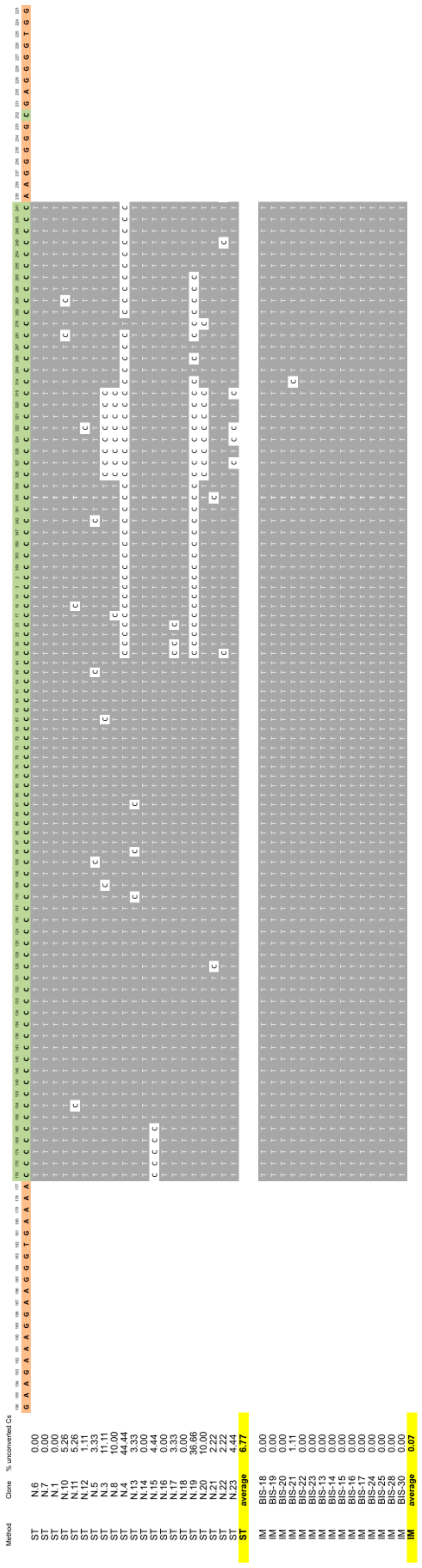

Figure S1

Figure S2

A

Method	Clone	% unconverted Cs
	REF	
ST	AS2	0.00
ST	AS.P.1	2.32
ST	AS.P.3	0.00
ST	AS.P.4	0.00
ST	AS.P.5	0.00
ST	AS.P.6	0.00
ST	AS.P.8	0.00
ST	AS.P.9	0.00
**ST**	**average**	**0.30**
IMP	AS+Bis1	0.00
IMP	AS+Bis2	0.00
IMP	AS+Bis8	0.00
IMP	AS+Bis9	0.00
IMP	AS+Bis7	0.00
IMP	AS+Bis6	0.00
IMP	AS+Bis5	0.00
IMP	AS+Bis3	0.00
IMP	AS+Bis4	0.00
IMP	AS+Bis11	0.00
**ST**	**average**	**0.00**

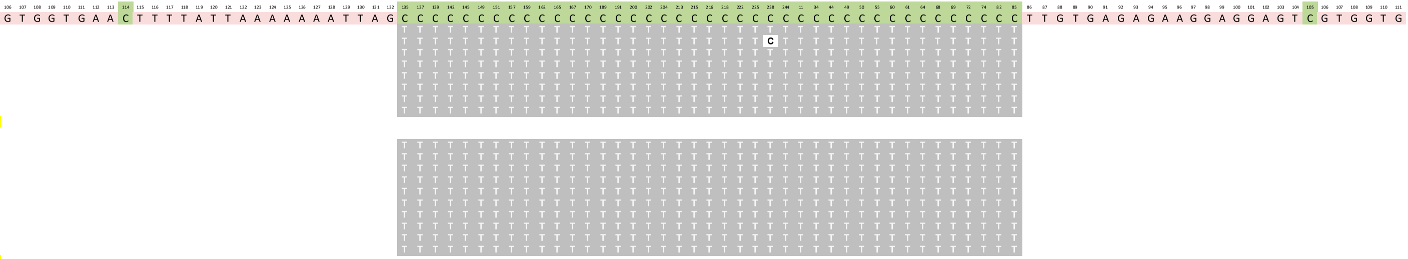

B

Method	Clone	% unconverted Cs
	REF	
ST	AS13	2.08
ST	AS.N.15	0.00
ST	AS.N.17	0.00
ST	AS.N.19	4.16
ST	AS.N.20	2.08
ST	AS.N.15	0.00
ST	AS.N.21	0.00
ST	AS.N.23	0.00
ST	AS.N.24	0.00
**ST**	**average**	**0.93**
IM	AS(-)Bis11	0.00
IM	AS(-)Bis9	0.00
IM	AS(-)Bis13	0.00
IM	AS(-)Bis12	0.00
IM	AS(-)Bis5	0.00
IM	AS(-)Bis8	0.00
IM	AS(-)Bis14	0.00
IM	AS(-)Bis6	0.00
**IM**	**average**	**0.00**

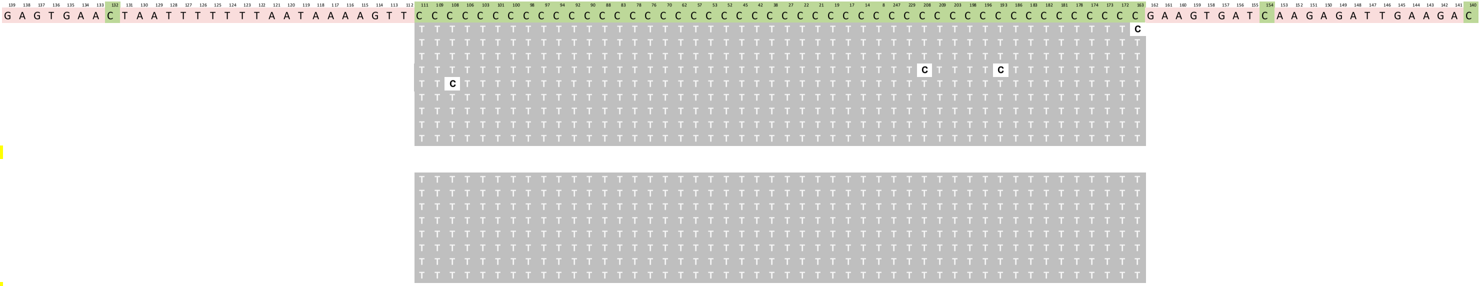

Method	Clone	% unconverted Cs
ST	HSVd-Met1	1.45
ST	HSVd-Met2	0.00
ST	HSVd-Met3	0.00
ST	HSVd-Met6	0.00
**ST**	**average**	**0.36**
IM	HSVd-Met15	0.00
IM	HSVd-Met18	0.00
IM	HSVd-Met19	0.00
IM	HSVd-Met8	0.00
IM	HSVd-Met20	0.00
IM	HSVd-Met21	0.00
IM	HSVd-Met22	0.00
IM	HSVd-Met23	0.00
IM	HSVd-Met24	0.00
IM	HSVd-Met25	0.00
IM	HSVd-Met26	0.00
IM	HSVd-Met27	0.00
**IM**	**average**	**0.00**

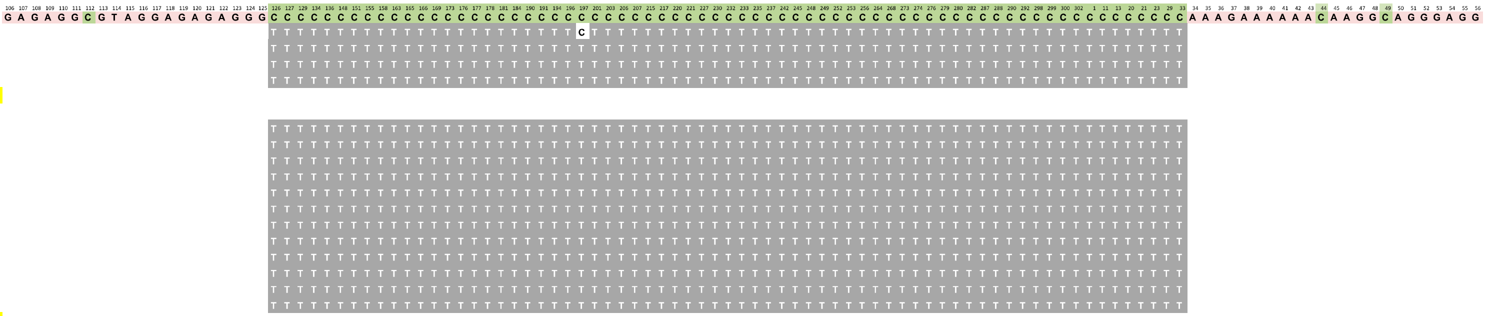

Figure S3
